# Eine Frage des Systems: Wie sich Shared Decision Making von selbst im Gesundheitssystem skaliert

**DOI:** 10.1007/s00103-025-04176-5

**Published:** 2026-01-08

**Authors:** Friedemann Geiger, Martin Oldenburg, Johannes Förner, Lars Mandelkow

**Affiliations:** 1 Nationales Kompetenzzentrum Shared Decision Making, Universitätsklinikum Schleswig-Holstein, Campus Kiel, Arnold-Heller-Straße 3, Haus 9, 24105 Kiel, Deutschland; 2 Medical School Hamburg, Hamburg, Deutschland; 3 Ministerium für Justiz und Gesundheit des Landes Schleswig-Holstein, Kiel, Deutschland; 4 Patientenbeirat Deutsches Krebsforschungszentrum, Heidelberg, Deutschland; 5 Ansgar University College, Kristiansand, Norwegen

**Keywords:** Kieler Modell, Synergetik, Selbstorganisation, Versorgungsqualität, Versorgungseffizienz, Kiel Model, Synergetics, Self-organisation, Quality of care, Cost efficiency

## Abstract

Shared Decision Making (SDM) ist in Deutschland noch kaum umgesetzt – ein Satz, mit dem sich buchstäblich seit Jahrzehnten jeder Artikel zum Thema SDM treffsicher eröffnen lässt. Aber warum? Mittlerweile wird SDM als Goldstandard zur Entscheidung über die bestmöglich passende Therapie angesehen. Das Patientenrechtegesetz unterstreicht diese Überzeugung. Wie kann es sein, dass sich SDM in der medizinischen Praxis dennoch nicht spürbar verbreitet?

In diesem Artikel wird unter Rückgriff auf die Synergetik beschrieben, welche Wirkkräfte des deutschen Gesundheitssystems für diesen Stillstand verantwortlich sind – und wie dieselben Wirkkräfte genutzt werden können, um SDM hervorzubringen. Als empirischer Beleg dieser Analyse zeigt das Kieler Modell, wie SDM mit dem SHARE-TO-CARE-Programm krankenhausweit implementiert und gemeinsam mit den Kostenträgern als eine erstattungsfähige Krankenkassenleistung operationalisiert wurde, deren positive Effekte bzgl. Versorgungsqualität und Kosteneffektivität eine sich selbst tragende und damit nachhaltige SDM-Versorgung ermöglichten.

Abschließend wird beschrieben, welche simplen Weichenstellungen durch den Gesetzgeber dafür sorgen würden, dass sich SDM nach dem Kieler Modell auch bundesweit selbstorganisiert in Krankenhäusern skaliert.

## Abstract (en)

Shared decision making (SDM) is still scarcely implemented in Germany—a statement that could have served as an appropriate opening line of literally any article on SDM during the last decades. But why? Today, SDM is regarded as the gold standard for reaching decisions about the most suitable treatment, a view reinforced by the German Patients’ Rights Act. How can it be that SDM nonetheless has not gained noticeable traction in medical practice?

Drawing on the framework of synergetics, this article outlines which factors within the German healthcare system are responsible for this stagnation and how those same factors can be utilized to foster the emergence of SDM. As empirical evidence for this analysis, the Kiel Model demonstrates how SDM was implemented hospital-wide through the SHARE TO CARE program and was operationalized as a reimbursable service covered by health insurance. Its positive effects on quality of care and cost-effectiveness enabled a self-sustaining and thus sustainable SDM infrastructure.

Finally, the article describes which simple regulatory adjustments by legislation would allow SDM to scale across hospitals nationwide through self-organization following the Kiel Model.

## Einleitung: Shared Decision Making ist rechtlich und ethisch geboten

In der Medizin werden täglich zahlreiche Entscheidungen getroffen. Neben eher unbedeutenden Entscheidungen der Arbeitsroutine gibt es auch viele mit weitreichender Bedeutung für Patienten und Behandelnde: Welche diagnostischen Maßnahmen sind notwendig, zu welchem Zeitpunkt? Welche Therapieoptionen sollen erprobt werden, in welcher Reihenfolge? Soll überhaupt jetzt behandelt werden oder ggf. später? Wie sind erhoffte Wirkung und mögliche Nebenwirkungen gegeneinander abzuwägen?

Während früher solche Entscheidungen von ärztlicher Seite allein getroffen wurden, herrscht heute Konsens, dass Arzt und Patient gemeinsam entscheiden sollten [1]. Diese gemeinsame Entscheidungsfindung wird im Englischen als Shared Decision Making (SDM) bezeichnet. Es ist zentraler Bestandteil patientenzentrierter Medizin [2].

In Deutschland wurde mit dem Patientenrechtegesetz 2013 festgelegt, wie medizinische Entscheidungen zu treffen sind. Hierfür wurde die damals meistzitierte SDM-Definition herangezogen, die von Charles et al. stammt [3]. Im § 630c Bundesgesetzbuch (BGB) heißt es: „Behandelnder und Patient sollen zur Durchführung der Behandlung zusammenwirken“. In § 630e wird im Einklang mit Charles et al. die Pflicht des Behandelnden benannt, „den Patienten über sämtliche für die Einwilligung wesentlichen Umstände aufzuklären“. Explizit wird ausgeführt: „Dazu gehören insbesondere Art, Umfang, Durchführung, zu erwartende Folgen und Risiken der Maßnahme sowie ihre Notwendigkeit, Dringlichkeit, Eignung und Erfolgsaussichten im Hinblick auf die Diagnose oder die Therapie“. Ebenfalls gemäß SDM-Definition wird betont: „Bei der Aufklärung ist auch auf Alternativen zur Maßnahme hinzuweisen, wenn mehrere medizinisch gleichermaßen indizierte und übliche Methoden zu wesentlich unterschiedlichen Belastungen, Risiken oder Heilungschancen führen können“. Dabei ist „Nichts tun“ bzw. „Abwarten und Beobachten“ unter Umständen ebenfalls eine „Alternative zur Maßnahme“.

Ohne den Begriff SDM explizit zu nennen, hat der Gesetzgeber damit die Anforderungen an medizinische Entscheidungsfindung so definiert, dass sie unter den bestehenden Modellen einzig von SDM erfüllt werden. Nur so kann von einer wirksamen Einwilligung des Patienten im Sinne von § 630d ausgegangen werden [1].

Die rechtlichen Vorgaben, so eindeutig sie auch sein mögen, sind lediglich Ausdruck eines noch gewichtigeren gesellschaftlichen Konsenses: Die Freiheit und Selbstbestimmung des Individuums sind zu schützen und zu fördern, auch und gerade angesichts gesundheitlicher Einschränkungen und in Situationen, in denen Abhängigkeit von Expertenwissen eine Rolle spielt. Hier ermöglicht SDM selbstbestimmte, informierte Entscheidungen als Ausdruck der Patientenautonomie und stellt damit einen auch aus ethischer Perspektive zu fordernden Wert an sich dar, weshalb man sie als eigenständigen patientenrelevanten Endpunkt im Sinne von § 39b Sozialgesetzbuch fünftes Buch (SGB V) betrachten kann [4].

Die Versorgungslandschaft in Deutschland spiegelt diesen Anspruch indes nicht wider. Zwar gab es in der Vergangenheit zahlreiche Aktivitäten zur Erhöhung des SDM-Levels in Deutschland – von einzelnen Förderprojekten über einen Förderschwerpunkt des Bundesministeriums für Gesundheit hin zur Integration von SDM in den Nationalen Kompetenzbasierten Lernzielkatalog Medizin (NKLM), um SDM bereits im Studium zu vermitteln [5]. Der Erfolg all dieser Bemühungen ist jedoch in der Fläche bescheiden, sowohl aus der subjektiven Sicht der Betroffenen als auch aus der objektiven Sicht externer Beobachter: Das SDM-Level ist insgesamt niedrig, niedriger als eine Klinik es leisten könnte und niedriger als Patienten es sich wünschen [6–8]. Das paternalistische Modell mit ärztlich dominierten Entscheidungen ist der Regelfall.

In diesem Artikel widmen wir uns daher zwei Fragen: Wie lässt sich diese Diskrepanz aus Anspruch und Wirklichkeit in Sachen SDM erklären? Dafür greifen wir zurück auf den systemtheoretischen Ansatz der Synergetik [9]. Daraus leiten wir gleichzeitig die Antwort auf die sich anschließende, noch wichtigere Frage ab: Wie kann eine bundesweite Implementierung von SDM gelingen?

## Systemimmanente Faktoren als Hemmnis für Shared Decision Making

Wie beschrieben wünschen sich Patienten mehr SDM [7]. Auch unter Ärzten herrscht schon seit Langem die Einsicht, dass SDM die bestmögliche Form der medizinischen Entscheidungsfindung ist [10]. Wenn SDM dennoch kaum umgesetzt wird, liegt es nahe, vor allem auf der Systemebene nach hemmenden Faktoren zu suchen.

Beim Verständnis von komplexen Systemdynamiken hilft die Perspektive der Synergetik [9]. Sie beschreibt, wie in einem System Übergänge von einem *stabilen Ordnungszustand* zu einem anderen stabilen Zustand erfolgen, in unserem Fall also der Übergang von einer Form der Entscheidungsfindung zu einer anderen. Die Persistenz des paternalistischen Entscheidungsmodells lässt sich dementsprechend als Ordnungszustand des Gesundheitssystems beschreiben, der in Kliniken und Praxen, aber auch in Verwaltungsvorgängen und in den Erwartungen einzelner Akteure verankert ist. Der Ordnungszustand ist *stabil*, weil es zahlreiche wirksame *Kontrollparameter* gibt. Darunter versteht man in der Synergetik Wirkfaktoren, die zum dauerhaften Fortbestehen des Ordnungszustands beitragen. Nachfolgend werden wesentliche Kontrollparameter aufgeführt, die bislang den Übergang von Paternalismus zu SDM behindern.

### Den Paternalismus stabilisierende Kontrollparameter

Ein entscheidender übergeordneter Kontrollparameter ist der ökonomische Druck im Gesundheitssystem, der sich auf mehrere Weisen auswirkt. Dieser Druck begünstigt eine Ausrichtung medizinischen Handelns auf Erlösoptimierung statt primär auf Patientenwohl. Arzt-Patienten-Gespräche *per se* sind vergleichsweise schlecht vergütet, weswegen sie geringe zeitliche und inhaltliche Priorität genießen. Zudem haben die gemäß den Anforderungen von SDM und Patientenrechtegesetz eigentlich gleichrangig darzustellenden Therapieoptionen teilweise sehr unterschiedliche Gewinnmargen: Wenn ein Neurochirurg im Krankenhaus dem Therapieentscheidungsgespräch beim Bandscheibenprolaps eine Operation folgen lassen kann, wird die Klinik in der Regel mit dem Eingriff Gewinn erwirtschaften. Einigen sich Arzt und Patient hingegen auf die Initiierung einer ambulanten Physiotherapie, wird die Klinik wegen der geringen Vergütung des Entscheidungsgesprächs an sich vermutlich mit diesem Patientenfall Verlust machen. Es besteht also ein ökonomischer Anreiz, Patienten im Zweifel eher zu einer umfangreicheren Therapie zu bewegen. Statt mit SDM herauszuarbeiten, was bestmöglich zum Patienten passt, wird finanziell belohnt, wer paternalistisch jene Therapie auswählt, die bestmöglich zum Leistungserbringer passt. Die Folgen sind Mengenausweitungen und Überversorgung – oder präziser: Fehlversorgung, wie sich bei zahlreichen Indikationen belegen lässt [11].

Vorgeschriebene Mindestmengen medizinischer Eingriffe in Behandlungszentren sind ein weiterer Kontrollparameter, der paternalistische Entscheidungsfindung zugunsten eines Eingriffs fördert. Während Mindestmengen aus Gründen der Qualitätssicherung zweifellos ihren Sinn haben, gefährden sie die ärztliche Offenheit für Patientenpräferenzen bzw. SDM allgemein insbesondere in jenen Einrichtungen, die die Mindestmengen ohne diesen Bias nicht erfüllen würden [11, 12].

Aus dem ökonomischen Druck resultiert darüber hinaus Zeitmangel beim Gesundheitspersonal, der seinerseits durch den Fachkräftemangel verstärkt wird. Zwar zeigen Studien, dass SDM nicht mehr Zeit erfordert als paternalistisches Entscheiden [13]. Zumindest während des Erlernens und Implementierens von SDM muss aber zunächst mehr Zeit investiert werden. Mit den neu erworbenen Gesprächsroutinen und verbesserter Gesprächsstruktur gelingt dann schnell mehr SDM in derselben Zeit [14]. Obschon also objektiv unbegründet, haben Ärzte ihrer subjektiven Einschätzung nach zu wenig Zeit für SDM [15].

### Überhangstabilität und Versklavung als weitere Veränderungshemmnisse

Die erwähnten suboptimalen SDM-Fertigkeiten bei vielen Ärzten sind ein weiterer hemmender Kontrollparameter. Diese stabilisieren sich selbst, denn gemäß dem *Law of the Instrument *werden Strategien insbesondere dann als passend angesehen und daher beibehalten, wenn man keine anderen kennt [16]. Dieser Effekt ist auch als *Maslows Hammer *bekannt: Wenn man nur einen Hammer hat, sieht alles wie ein Nagel aus [17].

Schon im Medizinstudium SDM zu vermitteln, um so mit der Zeit das SDM-Level im wahrsten Wortsinne heranwachsen zu lassen, ist im Prinzip eine gute Idee. Allerdings treffen Berufseinsteiger im Krankenhaus auf erfahrene Kollegen sowie auf ein System, in dem – wie oben beschrieben – wenig SDM vorkommt. Das Kompetenz- und das Hierarchiegefälle sorgen dafür, dass sich eher die Neuankömmlinge an das System anpassen als umgekehrt. Dass einzelne Systemelemente von der vorherrschenden Gesamtordnung in ebendiese hineingezwungen werden, kennt man aus anderen komplexen Systemen und wird in der Synergetik als *Versklavung* beschrieben [9]. Dabei fungieren die assimilierten Elemente in einer *zirkulären Rückkopplung *systemstabilisierend sowohl auf die vorhandenen als auch auf zukünftig hinzutretende Systemelemente. Versklavung und zirkuläre Rückkopplung erschweren somit Veränderungen hin zu mehr SDM.

Der letztgenannte Punkt verweist zudem auf eine generelle Eigenschaft etablierter Ordnungszustände: Sie weisen eine Veränderungsresistenz auf. Ordnungen können dadurch sogar noch fortbestehen, wenn sich die Kontrollparameter geändert haben, was in der Synergetik als *Überhangstabilität (Hysterese)* bekannt ist [9]. Das erklärt auch, warum in vergangenen Implementierungsstudien selbst prinzipiell wirksame, spezifische SDM-Interventionen ohne Wirkung bleiben, wenn die vom System aufgenommene „Dosis“ zu gering ist [18], und warum Systeme selbst im Falle initial erfolgreicher Interventionen nach deren Auslaufen wieder in den – paternalistischen – Ausgangszustand zurückfallen, sodass SDM nicht nachhaltig implementiert ist [19].

Zusammengefasst hilft die Synergetik also zu verstehen, warum sich Systeme *nicht* bewegen. Sie hilft aber auch zu verstehen, unter welchen Bedingungen dauerhafte Veränderungen möglich sind, wie nachfolgend beschrieben wird.

## Shared Decision Making ist alltagstauglich: Das Kieler Modell

Nach den obigen Ausführungen ist es fast überraschend, dass es im deutschen Gesundheitssystem dennoch funktionieren kann, SDM großflächig zu implementieren. Im Universitätsklinikum Schleswig-Holstein in Kiel wurde SDM zwischen 2018 und 2021 krankenhausweit eingeführt. Im Prä-Post-Vergleich stieg das SDM-Level sowohl subjektiv aus Patientensicht im Vergleich zur Kontrollgruppe [20] als auch objektiv aus der Sicht externer Beobachter [14]. In der Folge stieg die Gesundheitskompetenz von Patienten [20]. Bis heute wird die Implementierung von allen Kliniken aktiv aufrechterhalten und die genannten Endpunkte bleiben weiterhin signifikant erhöht [21].

Das Kieler Modell wurde nach den oben beschriebenen Prinzipien der Synergetik konzipiert. Daher bietet die Synergetik sich erneut zur Erklärung dieser Dynamik an: Warum ist in diesem Fall ein *Ordnungs-Ordnungs-Übergang* von Paternalismus zu SDM gelungen? Und warum ist der neue *SDM-Ordnungszustand* nachhaltig *stabil*?

Wenn man nicht dauerhaft gegen die oben beschriebenen SDM-hemmenden Kontrollparameter ankämpfen möchte, muss man deren Einfluss verringern. Zusätzlich empfiehlt es sich, wirksame SDM-fördernde Kontrollparameter hinzufügen. Nachfolgend werden beispielhaft einige der Kontrollparameter beschrieben, unter denen sich im Universitätsklinikum Schleswig-Holstein ab 2018 SDM herausgebildet hat.

### Das SHARE-TO-CARE-Programm zur krankenhausweiten Implementierung

Hier ist zunächst das SHARE-TO-CARE-Programm zu nennen [22]. Es wurde von einem multiprofessionellen Team im Rahmen eines Innovationsfondsprojekts gezielt für die krankenhausweite Implementierung von SDM entwickelt. Darin werden mehrere Module miteinander kombiniert, die bereits isoliert in randomisiert kontrollierten Studien ihre Wirksamkeit bewiesen haben. Damit werden alle relevanten Personengruppen im Krankenhaus adressiert – Ärzte, Pflegekräfte und Patienten. Konkret durchlaufen alle Ärzte ein fokussiertes Kommunikationstraining, alle Pflegekräfte werden geschult, wie sie den SDM-Prozess unterstützen können, und alle Patienten werden mit einer Awareness-Kampagne informiert, wie sie selbst aktiv ihre Beteiligung initiieren bzw. verstärken können. Zusätzlich werden ggf. Patientenpfade angepasst, um SDM auch strukturell zu ermöglichen. Um die verfügbare Informationsbasis zu den Therapieoptionen zu verbessern, stehen für die häufigsten Therapieentscheidungen jedes Fachgebiets evidenzbasierte Entscheidungshilfen online bereit.

Dabei wurde auf höchstmögliche Praktikabilität der Prozesse geachtet, sodass sie initial sowie langfristig kompatibel mit dem klinischen Alltag sind.

Mit der Krankenhausleitung, den Klinikdirektorien, dem Personalrat, dem Qualitätsmanagement und dem Changemanagement wurde die krankenhausweite Implementierung beschlossen und mit anderen Maßnahmen im Krankenhaus verzahnt.

Der Pioniercharakter in Sachen Patientenzentrierung wurde kontinuierlich journalistisch durch Print‑, TV- und Internetberichterstattung transportiert. Damit wurden sowohl Patienten innerhalb und außerhalb des Krankenhauses als auch Fachkräfte innerhalb des Krankenhauses adressiert. Dabei fungierten Fachkräfte und Patienten aus den zuerst implementierten Abteilungen als Testimonials und Fürsprecher für nachfolgende Kliniken.

An vielen Stellen des Implementierungsprozesses gab es zunächst – und gibt es vereinzelt heute noch – Skepsis, Widerstände und weitere Beharrungsbestrebungen. Längere Zeit schien der ursprüngliche Ordnungszustand schwer auflösbar, entsprechend der synergetischen *Überhangstabilität*. Über die Zeit und in der Summe aller Kontrollparameter gelang letztlich aber ein sich mit der Zeit beschleunigender, krankenhausweiter Kulturwandel – also ein Phasenübergang hin zum Ordnungszustand SDM: Das SDM-Level hatte sich signifikant über alle Abteilungen hinweg erhöht [14, 20]. Dies ist eine wichtige Lernerfahrung: Die Dosis der Kontrollparameter muss lang genug hoch genug sein, damit die Veränderung ohne Anstrengung fortbesteht.

### Die Rolle der Selbstorganisation bei der Implementierung von Shared Decision Making

Kontrollparameter erzwingen indes keine Ordnungen bzw. bestimmte Verhaltensweisen der Systemmitglieder. Sie bestimmen lediglich, welche Ordnungen möglich oder eher wahrscheinlich sind. Sie können SDM damit bestenfalls begünstigen. Ob SDM *tatsächlich* in einem konkreten Arzt-Patienten-Gespräch stattfindet, obliegt letztlich den handelnden Personen. Diese personenassoziierten Prozesse werden in der Synergetik als *selbstorganisiert *postuliert, d. h., sie entstehen aus sich selbst heraus ohne direkte Instruktion von außen [9]. Das erklärt auch, warum die Implementierung von SDM sich in jeder einzelnen Klinik in Kiel unterschiedlich entfaltete, obwohl das SHARE-TO-CARE-Programm überall mit denselben Bausteinen eingesetzt wurde. In manchen Kliniken ging die Leitung stärker mit eigenem Beispiel voran und sorgte für vollständige Umsetzung der Interventionen. In anderen Kliniken entstand der Prozess eher allgemein aus der Ärzteschaft heraus oder wurde stark von einzelnen Teammitgliedern angetrieben und inspiriert. In manchen Kliniken waren die Pflegekräfte proaktiv, in andern eher reaktiv. Auch Pull-Effekte von Patientenseite wirkten nicht in jeder Klinik gleichförmig oder gleichzeitig.

Gemeinsam ist indes allen in Kiel beobachteten Implementierungsprozessen, dass sie mit kleinen, instabilen Veränderungen im Denken und Handeln einzelner Beteiligter begannen. In der Synergetik spricht man in diesem Fall von *Fluktuationen*, die der Etablierung jeder neuen Ordnung vorausgehen. Wenn die Kontrollparameter dies ermöglichen, können sich diese kleinen Veränderungen gegenseitig verstärken im Sinne einer *zirkulären Rückkopplung.* Wenn etwa eine Ärztin das erste Mal ausprobiert, ihre Gesprächsstruktur so, wie im SDM-Training gelernt, zu gestalten, führt dies bei ihrem Patienten zu verändertem Verhalten, z. B. mehr Adhärenz, was wiederum die Ärztin bestärkt, zukünftig mehr SDM anzustreben. Patienten, die mehr SDM erwarten – etwa aufgrund der Berichte anderer Patienten oder durch Medienberichte über die SDM-Implementierung einer Klinik –, gehen aktiver in Gespräche hinein, womit sie es wiederum Ärzten erleichtern, mehr SDM zu zeigen usw. Zirkuläre Rückkopplungen können auf diese Weise die Wirkungen einzelner, isolierter Interventionsbausteine überproportional verstärken [9].

Das Prinzip der Selbstorganisation ist in diesem Zusammenhang auch deshalb so relevant, weil es ob der Komplexität des Gesamtsystems schlicht unmöglich wäre, SDM ausschließlich von außen zu verordnen und zu erwarten, dass die Mitglieder des Systems sich entsprechend verhalten. Formal kann man das Patientenrechtegesetz (§ 630 BGB) als eine solche Verordnung ansehen. Faktisch hat sich dadurch seit 2013 praktisch nichts geändert. Tatsächlich kennt kaum ein Arzt – geschweige denn ein Patient – dessen Vorgaben für medizinische Entscheidungen. Stattdessen muss es also darum gehen, einen Prozess in Gang zu setzen, bei dem SDM nicht als „ein weiteres Problem“, sondern als „die bestmögliche Lösung“ erkannt und von den Mitgliedern des Systems aktiv herbeigeführt wird.

Zusammenfassend griffen für die Einführung von SDM im Kieler Modell also von außen wirkende Kontrollparameter ineinander mit selbstorganisierten Anpassungen der Systemmitglieder selbst, in Wechselwirkung mit ihren Interaktionen untereinander.

### Shared Decision Making spart mehr ein, als es kostet

Die langfristige Aufrechterhaltung der Implementierung in den Kieler Kliniken wurde dadurch erleichtert, dass die Konsultationsdauer sich mit SDM nicht verlängerte; es gelang vielmehr, durch bessere Gesprächsstrukturierung mehr SDM in derselben Gesprächszeit zu realisieren [14]. Die Trainings neu eingestellter Fachkräfte konnten daher in die normale Facharztausbildung integriert werden.

Entscheidend bleibt aber sowohl bei der initialen Implementierung als auch bei der langfristigen Aufrechterhaltung von SDM, ob sich die aufzuwendenden zeitlichen und finanziellen Ressourcen für Trainings, Entscheidungshilfen, Patientenmaterialien sowie die kontinuierlichen strukturellen Optimierungen refinanzieren lassen. Zwar konnte die initiale Implementierung durch eine Förderung des Innovationsfonds finanziert werden. Die Aufrechterhaltung seit 2021 musste indes ohne weitere Fördergelder gelingen.

Erfreulicherweise konnte einerseits anhand von Daten der Techniker Krankenkasse belegt werden, dass mit SDM auch die Gesundheitskompetenz sowie in der Folge die Versorgungsqualität und Patientensicherheit anstiegen im Vergleich zu gematchten Kontrollpatienten aus den übrigen Krankenhäusern im Bundesgebiet. Konkret sank die Zahl der Notfalleinweisungen um 13 %. Parallel reduzierte sich der Ressourcenverbrauch im Krankenhaus, z. B. bei Bildgebungen. In der Summe sanken die Versorgungskosten für die Krankenkassen. Dabei waren die Einsparungen deutlich höher als die Kosten der Intervention. Die mit dem SHARE-TO-CARE-Programm ermöglichte Krankenhausleistung SDM ist also kosteneffektiv [14].

### Shared Decision Making als Krankenkassenleistung

Dieser Nachweis wiederum ermöglichte es, Krankenkassen davon zu überzeugen, SDM als eigenständige Leistung zu vergüten. Dafür wurde SDM als Regelungsinhalt eines Selektivvertrags nach § 140a festgeschrieben [23]. Als unbürokratische Operationalisierung triggert jeder Patientenfall ein pauschales Zusatzentgelt, wenn der Fall in einer Abteilung mit zertifizierter SDM-Implementierung erbracht wird. Die Entgelthöhe liegt deutlich unter den ermittelten Einsparungen pro Fall, ist aber gleichwohl kostendeckend für das Krankenhaus. Dafür erhalten bis heute alle Abteilungen ihre SDM-Implementierung aktiv aufrecht und lassen sich jährlich freiwillig rezertifizieren.

Ein zusätzlicher Kontrollparameter, der die langfristige Aufrechterhaltung monetär unterstützt, ist die mit SDM reduzierte Haftpflichtprämie des Krankenhauses. Dies begründet sich empirisch aus der erhöhten Patientensicherheit in Kiel in Kombination mit einer verringerten Klagewahrscheinlichkeit mit SDM [24].

## Die Skalierung des Kieler Modells im Gesundheitssystem

Zusammengefasst geht der Erfolg der krankenhausweiten SDM-Implementierung zurück auf die synergetisch fundierte Umsetzung im System Krankenhaus. Nachhaltig ist sie vor allem wegen der Nutzung eines Teils der SDM-assoziierten Einsparungen auf Krankenkassenseite für eine Vergütung von SDM, gepaart mit dem wiederholt kommunizierten positiven Erleben der Veränderung bei Patienten und Behandelnden. Diese Konstruktion ist Ausdruck gleichgerichteter Interessen von Krankenhaus und Krankenkassen – bei gleichzeitigem Nutzen für Patienten [23]. Wie lässt sich nun das Kieler Modell bundesweit skalieren?

Wie schon auf der Ebene des Krankenhaussystems gilt auf der Ebene des nationalen Gesundheitssystems, dass eine von außen vorgeschriebene Umsetzung von SDM – also gegen die im System wirkenden Kontrollparameter – kaum Erfolg haben dürfte. Stattdessen gilt es, den in Kiel erfolgten Prozess der Selbstorganisation in jedem deutschen Krankenhaus replizierbar zu machen.

Das betrifft zum einen die aktive Implementierung von SDM. Um dies zu ermöglichen, wurden dem Gemeinsamen Bundesausschuss (G-BA) nach Ende des Kieler Innovationsfondsprojekts alle Inhalte des SHARE-TO-CARE-Programms übergeben. Somit sind alle Bausteine frei verfügbar, die für die Erlangung des SHARE-TO-CARE-Zertifikats erforderlich sind. Die Zertifizierungskriterien sind dabei aufwärtskompatibel gestaltet: Werden zukünftig z. B. neue Trainingsmethoden für Ärzte entwickelt, die mindestens dieselbe Wirksamkeit aufweisen, können sie ebenfalls zur Erfüllung der Kriterien verwendet werden.

Angesichts der Effekte auf Versorgungsqualität und -effizienz hat der G‑BA dem Bundesgesundheitsministerium empfohlen, das Kieler Modell in die bundesweite Regelversorgung zu überführen [25]. Eine konkrete Umsetzungsmöglichkeit besteht darin, SDM als abrechnungsfähige Leistung ins Krankenhausentgeltgesetz aufzunehmen. Für die Definition der Leistung SDM kann der bestehende Selektivvertrag aus Kiel als Blaupause genutzt werden, über den bereits mehrere Hunderttausend Fälle abgewickelt wurden [23].

Die Regierungskommission für eine moderne und bedarfsgerechte Krankenhausversorgung [26] hat als weitere Umsetzungsoption vorgeschlagen, in Kliniken mit SHARE-TO-CARE-Zertifikat die mit dem Krankenhausversorgungsverbesserungsgesetz eingeführte Vorhaltepauschale aufzustocken.

In beiden Umsetzungsvarianten fungiert SDM mit Struktur- und Prozesszertifizierung als Qualitätsindikator. Analog funktionieren in Deutschland erfolgreich z. B. die Zentrumszuschläge nach § 136c Absatz 5 SGB V. Das pauschale Zusatzentgelt ermöglicht in zertifizierten Zentren dauerhaft höhere Versorgungsqualität. Gleichzeitig dient die Zertifizierung nach außen als Ausweis entsprechender Qualität und kann so von Zuweisern genauso als Auswahlkriterium genutzt werden wie von Patienten. Um diese Patientensteuerung zu unterstützen, hatte die Regierungskommission eine Eintragung der SDM-Zertifizierung im Bundes-Klinik-Atlas sowie weiteren Portalen empfohlen [26].

Die Empfehlungen von G‑BA und Regierungskommission zielen interessanterweise nicht auf eine Verpflichtung zur Implementierung von SDM ab. Sie setzen vielmehr darauf, dass innovative und patientenzentrierte Kliniken und Krankenhäuser aus Eigeninteresse SDM implementieren. Sie setzen lediglich die dafür nötigen Rahmenbedingungen, d. h. im synergetischen Verständnis die Kontrollparameter. Das Kieler Modell hat gezeigt, dass insbesondere die hier beschriebenen Kontrollparameter – die auch für eine bundesweite Skalierung empfohlen werden – ausreichen, um im Krankenhaus einen selbstorganisierten Prozess der Ordnungsbildung in Richtung von SDM als Standard medizinischer Entscheidungsfindung anzustoßen. [14].

## Fazit: Shared Decision Making kann sich von selbst bundesweit skalieren

Shared Decision Making bildet den Kern einer patientenzentrierten Versorgung [2]. Das Kieler Modell zeigt, dass synergetisch angelegte, krankenhausweite Implementierungen von SDM möglich sind. Mit dem gestiegenen SDM-Level erhöhten sich die Gesundheitskompetenz und die Patientensicherheit. Das zur Implementierung verwendete SHARE-TO-CARE-Programm spart dabei für die Krankenkassen mehr Geld ein, als es kostet [14]. Diese Kosteneffizienz von SDM zeigte sich analog in internationalen Anwendungen, wenn diese in ebenso großem Maßstab angelegt waren [27–29].

Das Kieler Modell belegt ferner, dass die dort verwendete struktur- und prozessorientierte Zertifizierung von SDM-Zentren ein pragmatischer sowie hinreichend valider Qualitätsindikator ist [14, 20]. Krankenkassen nutzen das SHARE-TO-CARE-Zertifikat daher als Voraussetzung für die spezifische Vergütung von SDM und fordern eine nationale Ausdehnung dieser Vergütungsmechanik durch den Gesetzgeber [23].

Eine bundesweite Skalierung von SDM braucht dabei keinen Zwang. Kostenträger, Leistungserbringer, Selbstverwaltung und Patienten haben ein gleichgerichtetes Interesse an SDM. Es müssen lediglich die Systembedingungen geschaffen werden, die einen intrinsisch angetriebenen, selbstorganisierten Roll-out im Sinne der Synergetik hervorbringen. Die notwendigen Systembedingungen sind durch das Kieler Modell definiert und können problemlos wahlweise ins Krankenhausentgeltgesetz oder ins Krankenhausversorgungsverbesserungsgesetz integriert werden [23, 25, 26].

In der Zukunft gilt es, auch für den ambulanten Sektor eine skalierbare SDM-Strategie zu entwickeln. Dafür kann vermutlich manches aus dem Kieler Modell übernommen werden, anderes muss für die spezifischen Anforderungen neu entwickelt werden. In jedem Fall dürften weitere Kosteneinsparungen zu erzielen sein bei Entscheidungen, wo Therapieoptionen nicht im Krankenhaus umgesetzt werden. In diesen Fällen wäre mit SDM eine Reduktion der Inanspruchnahme von entbehrlichen Krankenhausleistungen zu erwarten [27].

Das Vertrauen der Krankenhäuser auf die bundesweite Übertragung des Kieler Modells mit der korrespondierenden Abrechnungsfähigkeit von SDM führt bereits *vor* der gesetzlichen Verankerung zu regionaler Skalierung von SDM. Aktuell streben Kliniken in 10 Krankenhäusern an, das SHARE-TO-CARE-Zertifikat zu erlangen oder aufrechtzuerhalten. Mit dem Inkrafttreten der gesetzlichen Regelung wird sich diese Dynamik weiter beschleunigen und so mittels SDM gleichzeitig Patientenzentrierung, Versorgungsqualität und Versorgungseffizienz verbessern. Ein solch umfassender, bundesweiter Wandel wird Zeit brauchen. Aber jedes Krankenhaus, das im Zuge dieses Prozesses SDM implementiert, ist ein Gewinn für Leistungserbringer und Kostenträger – und vor allem für die Patientinnen und Patienten (s. Infobox).

### Infobox Kommentierung aus Patientensicht von Johannes Förner, Krebspatient und Mitglied im Patientenbeirat Krebsforschung des Deutschen Krebsforschungszentrums in Heidelberg

„Gemeinsame Entscheidungsfindung (SDM) bedeutet für Patienten, dass sie zusammen mit der Ärzteschaft ein gleichberechtigter Teil des Entscheidungsprozesses sind und dass ihre persönlichen Aspekte – Ziele, Ängste, Familie, Beruf, Lebensprioritäten – berücksichtigt werden. Bei vielen Erkrankungen gibt es nicht die eine ‚richtige‘ Entscheidung. Bei Krebserkrankungen zum Beispiel wägen Patienten häufig Überleben und Lebensqualität ab. Patienten, die an einem gemeinsamen Entscheidungsfindungsprozess teilnehmen, fühlen sich besser vorbereitet, zufriedener, und gehen selbstbewusster in die Therapie, an die sie sich dann auch halten. Dies hat nicht selten positive Auswirkungen sowohl auf den Therapieerfolg als auch darauf, wie man als Patient mit eventuellen Nebenwirkungen umgeht. Ich habe dies in einer klinischen Studie selbst erlebt.“
